# Quantitative Evaluation of Patch Test Reactions Using a 3D Camera-Derived Features and Machine Learning: The Role of Temporal Dynamics

**DOI:** 10.3390/bioengineering13070818

**Published:** 2026-07-16

**Authors:** Sotiria C. Gilou, Anna Tagka, George I. Lambrou, Emmanouil Papanastasiou, George K. Matsopoulos, Panagiotis D. Bamidis

**Affiliations:** 1 Lab of Medical Physics & Digital Innovation, Faculty of Medicine, School of Health Sciences, Aristotle University of Thessaloniki, AHEPA University General Hospital of Thessaloniki, 54636 Thessaloniki, Greece; gylousotir@auth.gr (S.C.G.); empapana@auth.gr (E.P.); 2 First Department of Dermatology and Venereology, “Andreas Syggros” Hospital, National and Kapodistrian University of Athens, 5 Ionos Dragoumi St., 11621 Athens, Greece; annatagka3@gmail.com; 3 Choremeio Research Laboratory, First Department of Pediatrics, National and Kapodistrian University of Athens, Thivon & Levadeias 8, 11527 Athens, Greece; glamprou@med.uoa.gr; 4 University Research Institute of Maternal and Child Health & Precision Medicine, National and Kapodistrian University of Athens, Thivon & Levadeias 8 Str., 11527 Athens, Greece; 5 Biomedical Engineering Laboratory, School of Electrical and Computer Engineering, National Technical University of Athens, Heroon Polytecnheiou 9 Str., Zografou, 15771 Athens, Greece

**Keywords:** allergic contact dermatitis, patch testing, machine learning, Antera 3D imaging, Random Forest, temporal changes

## Abstract

Background: Epicutaneous patch testing is the gold standard for diagnosing allergic contact dermatitis (ACD), yet its interpretation relies on subjective scoring and remains prone to inter-observer variability. Aim: In this study, we present a machine learning pipeline that complements subjective scoring with objective bioengineering measurements derived from the Antera 3D imaging system. Methods: A dataset of skin reactions was analyzed, with particular attention to how the data was split to avoid information leakage between patients. For this reason, methods such as GroupShuffleSplit and GroupKFold, which account for patient-level clustering, were used. Results: Of the models tested, the Random Forest classifier showed the best overall performance, with an AUC of 0.861 (95% CI: 0.830–0.888) on patient data that had not been used during training, outperforming the Multi-Layer Perceptron model. The incorporation of additional features that capture changes between 48 and 72 h improved the results even further, raising the AUC to 0.902 and achieving a very high sensitivity of 96.8%. Conclusions: Overall, the results show that objective biophysical measurements derived from the Antera 3D imaging system can be combined with machine-learning techniques for objective patch-test assessment. Incorporating temporal changes between the 48- and 72-h readings further improved model performance, suggesting that temporal changes provide additional information beyond single-time-point measurements.

## 1. Introduction

Allergic Contact Dermatitis (ACD) is a common inflammatory skin disease, affecting approximately 20% of the general population (overall prevalence 20.1%, 95% CI: 16.8–23.7%) and is a major problem in both clinical and occupational dermatology [1]. Early and accurate identification of the responsible allergens is crucial for effective disease treatment and relapse prevention. Patch tests are still considered the “gold standard” for ACD diagnosis, with reactions’ evaluation being carried out according to the International Contact Dermatitis Research Group (ICDRG) system, which is based on a four-point visual scale, ranging from negative (−) to strongly positive (+++) reaction [2].

Although the ICDRG scoring is widely used, it is based solely on the visual assessment of the examiner, which makes it quite subjective. Therefore, some degree of variation in expert judgments is to be expected. Differences have been observed in the way ICDRG criteria are applied [2], while several reports showed that there is also a significant discrepancy in the interpretation of patch tests, with an error rate of up to 24.6% in final assessments [3].

This issue becomes even more apparent in borderline cases, such as when it is essential to distinguish between a doubtful (?+) and a weakly positive (+) reaction. In these cases, uncertainty can directly affect the clinical decision. In case of inaccurate assessments the risk for false negatives or false positives increase. For example, false negative assessments could result to probable patient’s continuous exposure to potential allergens, or conversely, in case of false positives unnecessary avoidance instructions could be consulted and unnecessary burden on the patient’s daily life could be caused.

In recent years, there has been increasing interest in how artificial intelligence (AI) can be used for patch testing interpretation. Most studies report on convolutional neural networks (CNNs), which are applied either to clinical images or to processed color maps [4]. In some cases, data such as hemoglobin and texture maps from the Antera 3D imaging system are used [5], while other studies manifested good results even with simple clinical photographs [6]. It also appears that CNNs can contribute to the classification of reactions in patch tests [7]. However, although these results are encouraging, in most cases it is not easy to understand exactly how these models lead to automated decision-making. This makes their utilization difficult in a daily clinical practice, where concise interpretation of clinical outcomes is of major importance. Furthermore, the connection between imaging data and actual clinical significance is not always straight forward and often needs further confirmation or clarification.

In the present study, we propose a different approach, based not only on the health professional’s evaluation, but also on actual imaging data. More specifically, we utilized structured biomedical engineering features derived from the 3D multispectral imaging system Antera 3D. This system provides quantitative and reliable measurements of various skin properties, such as color (CIELAB) (CIELAB is derived from CIE L*A*B* = “colour space, which expresses color as three numerical values, L* for the lightness and a* and b* for the green–red and blue-yellow color components”), indices related to hemoglobin, surface roughness, but also volume, fluorescence and texture. This information is directly linked to clinically important elements, such as erythema, infiltration and surface morphology, and thus could potentially lead to improved skin reactions’ evaluation.

Thus, our approach included the transformation of imaging data to structured data tables, which could subsequently be used for further analysis, as for example in the present case, machine learning models, or standard statistical analysis. The proposed streamline, is easier to understand, can be consistently reproduced and, most importantly, could potentially be used in clinical practice.

Within this context, our study attempted to address some key questions; first, whether structured biomedical features can be used for a more objective patch-testing reactions classification. Second, we tried to address whether changes observed between 48 and 72 h affect diagnostic accuracy. Third, we investigated whether there is a correlation between specific allergens and the anatomical sites of previously diagnosed dermatitis. Finally, we investigated whether our results could be interpreted in such a way that could have critical meaning for clinical practice.

Overall, the present study aims at investigating if potential quantitative and more “transparent” measurements can function as a reliable, complement to traditional, more subjective diagnostic assessment.

## 2. Materials and Methods

### 2.1. Study Design and Data Collection

#### 2.1.1. Study Population

Details of the present study cohort have been previously reported, along with the followed methodology [5,8,9,10]. In short, the present study was designed as a prospective cohort study, including patients undergoing routine epicutaneous patch testing at the Laboratory of Patch Testing, National Referral Centre of Occupational Dermatoses, “Andreas Syggros” Hospital, National and Kapodistrian University of Athens, Greece.

Epicutaneous patch testing constitutes the gold standard diagnostic approach for the identification of causative allergens in allergic contact dermatitis (ACD), enabling controlled allergen exposure and standardized clinical assessment of delayed hypersensitivity reactions. A total of 200 patients with clinical suspicion of ACD were initially enrolled during the study period. Following data quality assessment, 194 patients met the criteria for completeness of the Antera 3D (Miravex Ltd., Dublin, Ireland) measurements and were included in the final machine-learning pipeline, yielding 4477 observations. The remaining six patients were excluded because of incomplete Antera 3D measurements, such as image acquisition failure at critical time points or technical camera-related issues. These exclusions were technical in nature and were not related to patients’ clinical characteristics. Patients were consecutively recruited from those referred during the study period.

#### 2.1.2. Inclusion and Exclusion Criteria

Patients were admitted to the Laboratory of Patch Testing, National Referral Centre of Occupational Dermatoses, and were evaluated for suspected contact dermatitis [11,12]. In case of factors that could influence skin reactions interpretation, patients were excluded. In particular, patients were excluded if they were under: systemic immunosuppressive therapy [13], chronic corticosteroids [14], or anti-inflammatory therapy [15]. In addition, subjects with chronic dermatological diseases [14] and those with recent severe UV exposure [16] were also excluded. Patients for which data collected with the Antera 3D system were either incomplete or missing were also excluded.

#### 2.1.3. Patch Testing Protocol

The European Baseline Series includes several allergen categories, such as metals, fragrances, preservatives, rubber materials, topical medications, and other ingredients associated with allergic contact dermatitis [8,9,10,17]. Patch tests were performed with a standardized panel, which includes approximately 30 of the most common allergens, such as metals, preservatives, fragrances, and acrylics. Allergens were applied to the upper back with standard patches and left on for 48 h. Assessments were made at 48 and 72 h after application. Final assessment was performed at 72 h.

#### 2.1.4. ICDRG System

Skin reactions were independently scored by experienced health professionals according to the ICDRG guidelines. Scoring was based on features such as erythema, infiltration, papules and vesicles, on a scale from negative (0) to strongly positive (+++). Although widely used, this method remains somewhat subjective, especially in borderline cases. For the purpose of analysis, results were summarized into two categories: reactions with a score of ≥1 were considered positive, while those with a score of 0 were considered negative.

#### 2.1.5. Antera 3D Imaging and Bioengineering Features

Quantitative assessment of skin reactions was performed using the Antera 3D imaging system (Miravex Ltd., Dublin, Ireland), which allows for the three-dimensional imaging of skin and the measurement of parameters such as hemoglobin and melanin. Parameter sampling was performed with Antera 3D software (Miravex Ltd., Dublin, Ireland, version 1.0). Measurements were performed at three time points (24, 48, and 72 h) to monitor the progression of skin reaction over time. A total of 41 biomedical features were extracted, which were divided into six main categories: color parameters (CIELAB: L*, a*, b*, and ITA), hemoglobin-related indices for assessing erythema, roughness measurements describing skin texture, volumetric features related to papule elevation, fluorescence features, and indices describing skin folds and structural changes (Table 1). These characteristics are linked to key clinical features, such as the severity of erythema, infiltration and changes in skin appearance. At the same time, demographic and anthropometric patient data, such as age, gender, smoking, allergen, site of occurrence and relevant history, were also collected.

#### 2.1.6. Ethics Statement

The present study was approved by the Institutional Scientific Review Board of the University Hospital “Andreas Syggros”, National and Kapodistrian University of Athens, Medical School (Protocol. Nr. 2851/2018), and the ethical considerations were fully consistent with the Declaration of Helsinki (1975, review 2000). The data were kept anonymously, and there is no way to track back to the patient’s personal data. Prior to inclusion, all participants provided written informed consent.

### 2.2. Data Preprocessing and Feature Engineering

Clinical, biomedical, photonics and patient data were merged into a single data matrix. Missing values in continuous variables were handled using k-nearest neighbors (KNN) imputation with k = 5 and distance-based weighting. This method preserves relationships between data and avoids errors that can arise from simpler techniques.

Some basic corrections were also made to make the data more consistent. For example, when volumetric measurements were zero, the corresponding height values were also set to zero to be logically consistent. Categorical variables were converted to binary (0, 1) when necessary. In addition, occupational risk was categorized and some clinical variables were adjusted so that they could be used correctly in machine learning models.

### 2.3. Dataset Structure and Splitting Strategy

The final dataset included 4477 observations from 194 patients, as each patient contributed multiple measurements across different allergens and time points. To avoid information leakage arising from repeated measurements from the same individual, all data partitions were performed at the patient level. The dataset was initially divided into training (80%) and test (20%) sets using grouped splitting, ensuring that all observations from a given patient were assigned exclusively to one of the two sets. This resulted in approximately 3494 observations from 155 patients in the training set and 983 observations from 39 patients in the independent test set. Consequently, model performance was assessed on patients not encountered during model development.

Within the training set, model performance was evaluated using 5-fold cross-validation, again at the patient level. All observations from the same patient were assigned to the same fold, preventing overlap between training and validation subsets. The validation folds were of comparable size, containing 31, 31, 31, 32 and 30 patients, respectively (approximately 700 observations each), which together account for all 155 training patients. The independent test set (39 patients; 983 observations) was held out completely and did not participate in the cross-validation procedure. Cross-validation performance metrics are reported as the mean and standard deviation across the five validation folds.

To further minimise the risk of data leakage, variables directly derived from the outcome labels were excluded before model training. In addition, all preprocessing steps that learned from the data, including KNN imputation, feature scaling where applicable, and SMOTE oversampling, were incorporated within the modelling pipeline and fitted exclusively on the training folds before being applied to the corresponding validation data.

### 2.4. Synthetic Minority Over-Sampling Technique (SMOTE)-Based Class Imbalance Mitigation

The data was unbalanced, with fewer positive responses than negative ones. To correct this, we used the SMOTE method on the training data, which creates new similar samples for the less frequent category. In the training set, this corresponded to 600 positive (17.2%) and 2894 negative (82.8%) observations before resampling; after SMOTE, the minority class was oversampled to 2894 positive and 2894 negative observations (5788 in total). This procedure was only applied to the training set, in order to leave unaffected the overall evaluation. Where possible, we adjusted the category weights in training.

### 2.5. Machine Learning Models

All machine-learning analyses were implemented using Python version 3.14.0. Data preprocessing and management were performed using pandas version 3.0.0 and NumPy version 2.4.2. Machine-learning models and evaluation were implemented using scikit-learn version 1.8.0, while SMOTE-based resampling was performed using imbalanced-learn version 0.14.1. The multi-layer perceptron (MLP) was implemented using PyTorch version 2.10.0, gradient boosting was performed using XGBoost version 3.2.0, and model interpretability was conducted using SHAP version 0.50.0.

Random Forest was used as the main model, because it can handle different types of data and capture more complex relationships without “learning by heart”. Other models were also tested for comparison, such as logistic regression and a neural network (Multi-Layer Perceptron (MLP)). At the same time, the intensity of the reaction was examined with XGBoost, in order to obtain a more accurate evaluation of the severity on the ICDRG scale. Especially, the MLP utilization included the following features: Architecture: (a) Three fully connected hidden layers with a pyramidal width of 128, 64, and 32 neurons, (b) Each hidden block consisted of Linear, BatchNorm1d, GELU activation, and Dropout (*p* = 0.3), (c) the output is a single linear unit returning a logit, paired with BCEWithLogitsLoss and a pos_weight term to compensate for class imbalance (no terminal sigmoid, for numerical stability) and (d) Input dimensionality is approximately 50 bioengineering features. Training configuration: (a) Optimizer: AdamW (learning rate 1 × 10^−3^, weight decay 1 × 10^−4^) with ReduceLROnPlateau (factor 0.5, patience 10, min_lr 1 × 10^−6^, (b) Batch size 64, maximum 200 epochs, early stopping with patience of 25 epochs on validation loss, and gradient clipping at max_norm = 1.0 and (c) Patient-level GroupShuffleSplit (80/20) for train/test and patient-level GroupKFold (k = 5) for cross-validation, with SMOTE and StandardScaler fitted only on the training fold to prevent leakage. The reported test prediction is the ensemble average across the five fold models (random seed = 42). Design rationale: BatchNorm stabilizes training and reduces sensitivity to learning-rate selection, Dropout 0.3 prevents co-adaptation of neurons on a dataset of moderate size (*n* approximately 4500 observations from 194 patients), and GELU was chosen for its smoother gradient behavior than ReLU on small networks [18]. AdamW was preferred over Adam because of its decoupled weight decay, which is better behaved as an L2 regulariser.

Hyperparameter selection differed according to model complexity. For the KNN baseline, hyperparameters were optimised using GridSearchCV under patient-level GroupKFold cross-validation. In contrast, the Random Forest parameters (n_estimators = 200, max_depth = 10, and class_weight = “balanced”) and the MLP architecture were predefined based on commonly used configurations for tabular medical datasets of comparable size.

### 2.6. Temporal Feature Engineering

In order to examine how skin reactions evolve over time, features (temporal delta) that record changes between measurements’ intervals were utilized. For each bioengineering feature, the difference between 72 h and 48 h measurements was calculated as:

Δ*f* = *X*(*f*, 72 h) − *X*(*f*, 48 h) (1)

These characteristics show how quickly and towards which direction inflammation changes. In other words, allergic reactions usually increase over time, while irritant reactions tend to decrease. This way, the model can more easily distinguish between these two cases.

### 2.7. Model Interpretability

Model interpretation was performed using the SHapley Additive exPlanations (SHAP) method, which detects the contribution of each feature to the predictions. For all tree models, the TreeExplainer algorithm was used, which allows for fast and reliable calculation of these contributions. The analysis was performed on the independent test set, so that the results reflect the behavior of the model on new data and not on the training data. That way, predictions can be interpreted based on clinically important features, enhancing transparency and facilitating their use in practice.

### 2.8. Statistical Analysis

Model performance for binary classification was mainly evaluated using the Area Under the Curve (AUC) methodology. At the same time, other metrics were calculated, such as sensitivity, specificity, positive and negative predictive value (PPV, NPV), F1-score and confusion matrices, so that the evaluation is closer to clinical practice.

**Figure 1:**
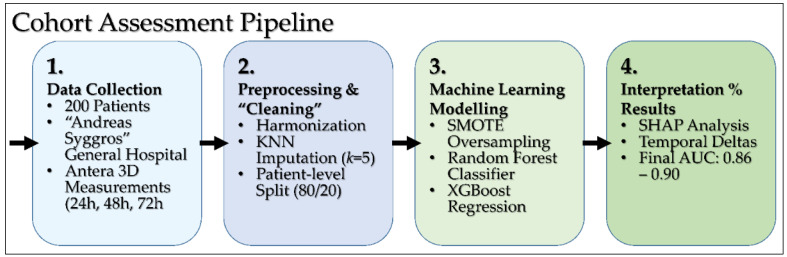
Overview of the study workflow. Following data collection and acquisition of Antera 3D measurements from patients undergoing routine patch testing, preprocessing procedures were applied, including harmonisation, imputation, and patient-level train–test splitting. Machine learning models were subsequently developed and evaluated, and their outputs were explored using SHAP analysis and temporal analyses to identify the most informative predictors of patch test reactions.

Root Mean Square Error (RMSE) and the coefficient of determination (*R*^2^) were used to estimate the severity. All metrics were calculated with bootstrap (2000 repetitions). To ensure reproducibility, a fixed random value (random_state = 42) was used in the primary analyses, including data partitioning, balancing, and model training. The entire process was performed in a repeatable manner so that the same steps were applied each time (Figure 1). In addition, a sensitivity analysis using multiple random seeds (42–51) was conducted to assess the stability of the Random Forest model, and the corresponding results are presented in Supplementary Table S1.

Statistical analyses were performed using SciPy version 1.17.0 and statsmodels version 0.14.6.

## 3. Results

### 3.1. Cohort Characteristics

In total, 200 patients were included in the present study. After data review and removal of incomplete records, 194 patients were included in the final analysis. The data set included 4477 observations, reflecting multiple allergen–time point measurements per patient. Out of the 194 patients, 155 (79.9%) were female and 39 (20.1%) were male. The mean age was 41.9 ± 13.7 years. Regarding smoking, 113 patients (56.5%) were non-smokers, 68 (34.0%) were smokers and 13 (6.5%) were former smokers.

Atopic dermatitis (AD) was recorded in 80 patients (41.2%). The most frequent location of dermatitis was on the hands (59.5%), followed by the face (36.0%), the trunk (22.0%) and the lower extremities (16.0%). Occupational dermatitis was observed in 34.5% of all patients. At the observational level, the data were unbalanced, as 17.4% of the reactions were positive and 82.6% negative. For this reason, methods to address the imbalance were applied during the development of the model (Section 2.4). The main demographic and clinical characteristics are presented in Table 2.

### 3.2. Performance of the Main Binary Classification Models

Τhree machine learning models were trained and compared: Random Forest, logistic regression, and multilayer perceptron (MLP) in order to evaluate model performance (Section 2.5). Random Forest showed the highest discrimination ability in the independent test set, with an AUC of 0.8611. Logistic regression and MLP followed with slightly lower AUC values (0.8437 and 0.8365, respectively). Although differences in AUC were similar, more substantial differences were observed in clinical metrics. Specifically, Random Forest had the highest sensitivity (67.2%), thus it was more efficient at identifying positive reactions. In contrast, logistic regression showed the highest specificity (90.7%), but with a lower sensitivity (61.6%). MLP showed intermediate performance, without excelling in any metric.

Cross-validation results confirmed the stability of these findings, with mean AUC values of 0.8691 ± 0.0171 for Random Forest, 0.8729 ± 0.0173 for logistic regression, and 0.8258 ± 0.0200 for MLP. Despite similar AUCs between Random Forest and logistic regression, Random Forest showed a better balance between sensitivity and specificity in the independent test set. Thus, Random Forest was selected as the base model for further analysis. The results are summarized in Table 3.

Despite similar AUCs between Random Forest and logistic regression, Random Forest showed a better balance between sensitivity and specificity in the independent test set. Thus, Random Forest was selected as the base model for further analysis. The results are summarized in Table 3.

Additional benchmarking against state-of-the-art tabular models (XGBoost and LightGBM) is provided in Supplementary Table S2.

### 3.3. Effect of Class Imbalance Mitigation

To assess the effect of class imbalance (Section 2.4), a sub-analysis was performed with the Random Forest model, testing three different training settings: (i) using SMOTE together with balanced class weights, (ii) only balanced class weights without SMOTE, and (iii) without any balancing method.

The configuration combining SMOTE and class weighting achieved a test AUC of 0.861, with sensitivity of 67.2% and specificity of 86.1%. Removing SMOTE while retaining class weights resulted in a slight increase in AUC (0.878) and specificity (90.7%), but reduced sensitivity (61.6%). In the absence of any balancing strategy, the model achieved the highest AUC (0.889) and specificity (97.5%), but sensitivity decreased substantially to 37.3%.

These results highlight a clinically important trade-off. Although higher AUC values were observed in unbalanced settings, the ability to detect positive reactions was markedly compromised. Given that the primary objective is to identify true allergic reactions, sensitivity is of critical importance.

**Figure 2:**
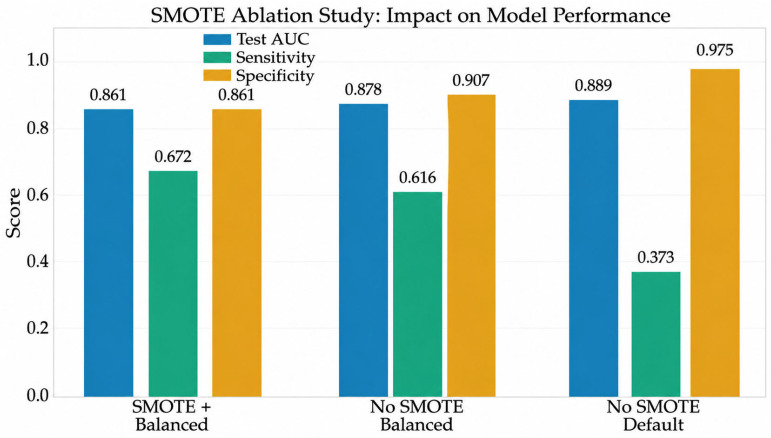
Effect of class imbalance handling strategies on model performance. The SMOTE-based configuration provides the most balanced trade-off between sensitivity and specificity.

Accordingly, the SMOTE-based configuration was selected as the final training strategy, as it provided the most appropriate balance between sensitivity and specificity for the intended clinical application. The results are illustrated in Figure 2.

### 3.4. ROC Analysis, Confusion Matrix and Threshold Optimization

**Figure 3:**
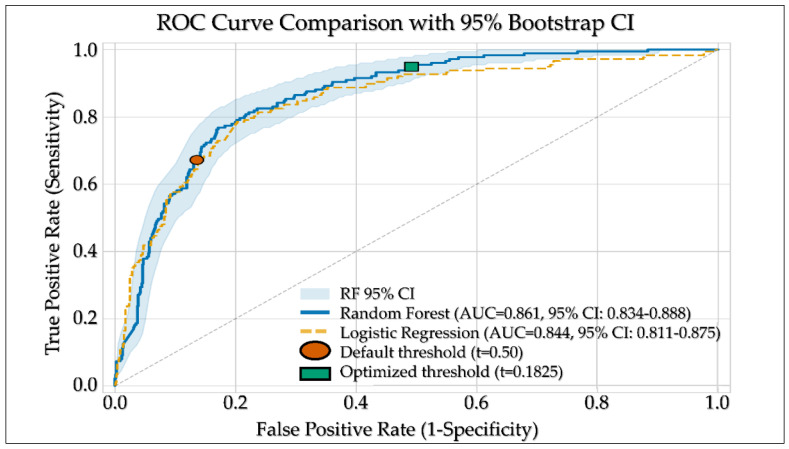
ROC curve of Random Forest on the test set, with 95% CI (AUC = 0.8611, 95% CI: 0.834–0.888).

The discriminative performance of the final Random Forest model was evaluated using Receiver Operating Characteristic (ROC) analysis. The model achieved an AUC of 0.8611 (95% CI: 0.8301–0.8880) on the independent test set (Figure 3), indicating strong ability to distinguish between positive and negative reactions.

**Figure 4:**
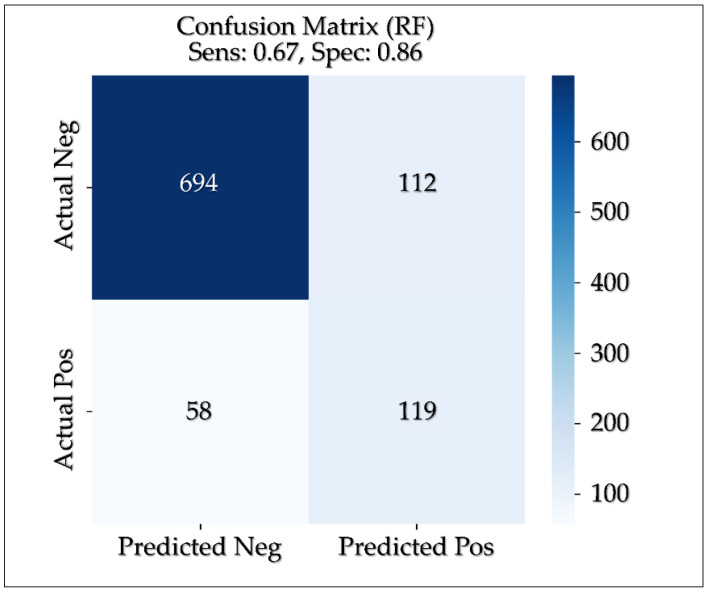
Confusion matrix for the Random Forest model on the test set (*n* = 983 observations, 39 patients). TN = 694, FP = 112, FN = 58, TP = 119. Sensitivity = 67.2%, specificity = 86.1% at threshold *t* = 0.50.

The cross-validation performance (CV AUC = 0.8691 ± 0.0171) was close to that of the test set, indicating stable performance on unseen data, without apparent overfitting. In the test set (*n* = 983 observations from 39 patients), 694 true negatives (TN), 112 false positives (FP), 58 false negatives (FN), and 119 true positives (TP) were recorded by the model (Figure 4). With the default threshold (*t* = 0.50), the sensitivity was 67.2% and the specificity was 86.1%. Bootstrap-derived 95% confidence intervals were additionally calculated for the main performance metrics. Sensitivity was 0.672 (95% CI: 0.599–0.743), specificity was 0.861 (95% CI: 0.836–0.885), positive predictive value (PPV) was 0.515 (95% CI: 0.449–0.581), and negative predictive value (NPV) was 0.923 (95% CI: 0.902–0.942).

With a lower threshold (*t* = 0.1825), the sensitivity increased to 94.9%, while the specificity decreased to 64.7%. False negatives were reduced from 58 to 9, which significantly improves the detection of true positives. This is considered acceptable in screening cases in which it is preferable to identify more positive cases, even if false positives increase (Figures S1a and S1b).

To further characterize model behavior during cross-validation, the confusion matrix aggregated over the five held-out validation folds is reported in Table 4. Because SMOTE was applied only to the training portion of each fold, every held-out fold retained its original, non-resampled class distribution, so the matrix reflects performance on real (non-synthetic) observations.

The corresponding pooled metrics were sensitivity = 0.717, specificity = 0.858, and precision = 0.511, consistent with those obtained on the independent test set (Figure 4) and indicating stable performance across the two evaluation schemes.

### 3.5. Model Interpretability (SHAP Analysis)

**Figure 5:**
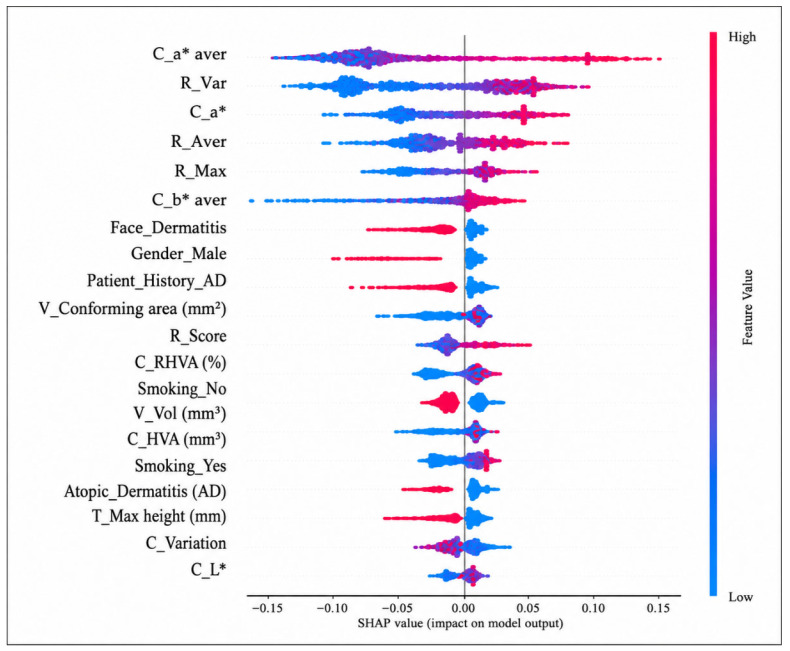
SHAP summary (beeswarm) plot of the 20 most influential features for the Random Forest model on the independent test set. Each point represents one observation; its position on the horizontal axis is the SHAP value (impact on the model output) and its colour encodes the feature value (blue = low, red = high). Features are ordered from top to bottom by mean absolute SHAP value. Legend: C_a* aver: mean a* component (CIELAB red-green axis) within the selected region; R_Var: spatial variation of haemoglobin concentration; C_a*: red-green CIELAB colour component; R_Aver:average haemoglobin concentration; R_Max: maximum haemoglobin concentration; C_b* aver: mean CIELAB b* (yellow-blue) component; Face_Dermatitis (FD): dermatitis on the face; Gender_Male: male subjects; Patient_History_AD: history of atopic dermatitis; V_Conforming area (mm^2^): surface area contributing to volume calculation; R_Score: haemoglobin-related severity; C_RHVA (%): relative high-variation area; Smoking_No: non-smoker; V_Vol (mm^3^): total volume of surface deviations; C_HVA (mm^2^): high colour-variation area; Smoking_Yes: smoker; Atopic_Dermatitis (AD): presence of atopic dermatitis; T_Max height (mm): maximum surface height; C_Variation: colour heterogeneity; C_L*: CIELAB lightness (skin brightness).

The model was interpreted using the SHAP method to examine which features have the greatest impact on the predictions. As shown in Figure 5, the most important features are mainly related to erythema and color parameters, such as C_a aver*, C_a*, R_Aver, R_Max and R_Var (Figure 5). C_a aver* (average a* value in the CIELAB space) seems to have the greatest impact, as it expresses the degree of redness. R_Aver and R_Max correspond to the average and maximum intensity of erythema, while R_Var shows how uniform its distribution is on the skin. These findings are consistent with the clinical practice, where the intensity and distribution of erythema play a key role in the assessment of reactions. The fact that the model is mainly based on these features shows that its predictions have a reasonable basis and can be interpreted.

An additional SHAP-guided feature ablation analysis demonstrated that reduced feature sets achieved performance comparable to the full model; the corresponding results are presented in Supplementary Table S3.

In addition to these expected drivers, several non-morphological variables also contributed to the model predictions. Features such as sex, history of atopic dermatitis, anatomical location, and smoking status may reflect differences in baseline reactivity, exposure patterns, or residual confounding rather than direct biological mechanisms. Their contribution should therefore be interpreted with caution.

A comparison of SHAP rankings between the baseline and temporal models revealed that several erythema-related descriptors remained consistently important, including C_a*, C_a* aver, R_Aver, R_Max, R_Var, and V_Conforming area, suggesting a stable core of predictive features (Figures S2 and S3). At the same time, temporal delta descriptors emerged among the most influential predictors in the temporal model. In particular, Delta_R_Var may reflect temporal changes in the spatial heterogeneity of erythema distribution, whereas Delta_R_Score may capture changes in the overall intensity of erythema between 48 and 72 h. These observations suggest that the temporal evolution of the skin response provides complementary information to static morphological characteristics, although its relationship with routine clinical assessment requires further investigation.

The stability of SHAP-derived feature rankings was further assessed across the five patient-level GroupKFold validation folds. The complete per-fold rankings and their visual representation are provided in Supplementary Tables S4 and S5 and Figure S4, respectively, demonstrating consistent identification of the most influential predictors within the study cohort.

### 3.6. Temporal Dynamics Improve Diagnostic Performance

To evaluate the contribution of temporal data (Section 2.6), various combinations of features were considered when training the models. The best performance was achieved by the model that incorporated 72-h measurements in combination with the time delta (Δ = 72 h − 48 h), achieving an AUC of 0.902. This value is significantly superior to the basic model (AUC = 0.861), which lacked explicit temporal coding.

At the screening level, the sensitivity reached 96.8%, with only three false negatives out of a total of 93 positive samples in the test subset. The best result shows how important it is to consider the evolution of reactions over time. In practice, allergic reactions usually become more intense, while irritant reactions tend to decrease. Recording these changes helps the model better distinguish between the two cases.

Beyond the overall AUC improvement, the individual temporal configurations are listed comparatively in Table 5. The incorporation of temporal delta features (72 h + Δ) yielded systematically better results, enhancing both the discrimination and sensitivity levels. For the predefined threshold (*t* = 0.50), the sensitivity ranged between 63.8% and 71.0% across all models, while the specificity remained high, ranging from 81.2% to 88.6%. The temporal delta model achieved the highest sensitivity (71.0%) while maintaining high specificity (86.2%).

Under screening conditions (*t* = 0.22), sensitivity increased across all models but was highest for the temporal delta configuration (96.8%), compared to 85.7–93.5% for the remaining models. As expected, specificity decreased (47.7–60.7%), reflecting the trade-off inherent to screening scenarios. Overall, the temporal delta model identified nearly all positive reactions, with minimal increase in false positives, supporting its potential utility as a clinical decision-support tool.

### 3.7. Allergen–Location Associations

**Figure 6:**
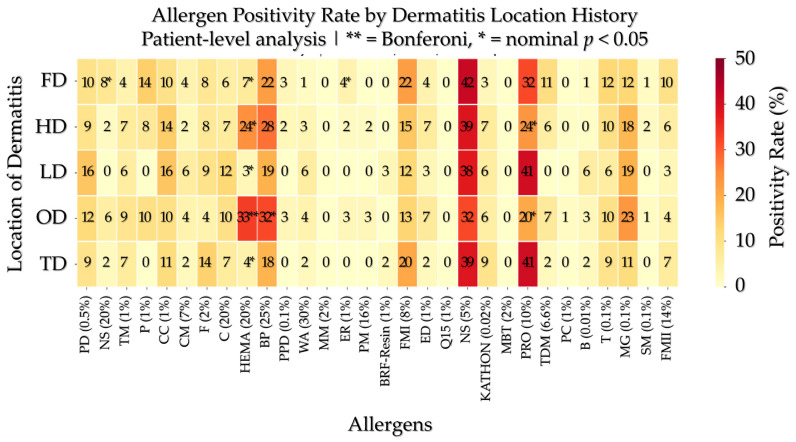
Heatmap of allergen–location associations. Color intensity represents positivity rates. Only the association between HEMA and occupational dermatitis remains statistically significant after Bonferroni correction (**Legend**: **FD**: Face Dermatitis, **HD**: Hand Dermatitis, **LD**: Leg Dermatitis, **OD**: Occupational Dermatitis, **TD**: Trunk Dermatitis. **PD (0.5%)**: Potassium Dichromate 0.5%, **NS (20%)**: Neomysin Sulphate 20%, **TM (1%)**: Thiuram Mix 1%, **P (1%)**: Paraphenylenediamine 1%, **CC (1%)**: Cobalt Chloride 1%, **CM (7%)**: Caine Mix 7%, **F (2%)**: Formaldehyde 2%, **C (20%)**: Colophonium 20%, **HEMA (20%)**: 2-Hydroxyethyl -Methacrylate(HEMA) 2%, **BP (25%)**: Balsam of Peru 25%, **PPD (0.1%)**: PPD Black Rubber Mix 0.1%, **WA (30%)**: Wool Alcohols 20%, **MM (2%)**: Mercapto Mix 2%, **ER (1%)**: Epoxy Resin 1%, **PM (16%)**: Paraben Mix 16%, **BRF-Resin (1%)**: Paratertiarybutyl Phenol formaldehyde Resin, **FMI (8%)**: Fragrance Mix I, **ED (1%)**: Ethylenediamine Dihydrate 1%, **Q15 (1%)**: Quaternium 15%, **NS (5%)**: Nickel Sulphate 5%, **KATHON (0.02%)**: 5-Chloro-2-methyl-4-isothiazolin-3-one/2-Methyl-4-isothiazolin-3- one 0.02%, **MBT (2%)**: Mercaptobenzothiazole 2%, **PRO (10%)**: Propolis 10%, **TDM (6.6%)**: Textile Dye Mix 6.6%, **PC (1%)**: Petroleum Control 1%, **B (0.01%)**: Budesonide 0.01%, **T (0.1%)**: Thiomersal 0.1%, **MG (0.5%)**: Methyldibromo-Glutaronitrile 0.5%, **SM (0.1%)**: Sesquiterpenelactone Mix 0.10%, **FMII (14%)**: Fragrance Mix II 14%).

To explore potential clinical patterns beyond prediction (Section 2.8), allergen–location associations were evaluated using Fisher’s exact test. A total of 150 associations (30 allergens across 5 dermatitis locations) were examined, and Bonferroni correction was applied to account for multiple comparisons. Twelve associations were statistically significant before correction (*p* < 0.05); however, only one remained significant after adjustment. The strongest and most robust association was observed between 2-hydroxyethyl methacrylate (HEMA) and occupational dermatitis. HEMA positivity was observed in 33.3% (23/69) of patients with occupational dermatitis compared with 8.4% (11/131) of those without occupational dermatitis (*p* < 0.0001; Bonferroni-corrected *p* < 0.001). The remaining associations did not retain statistical significance after correction and should therefore be interpreted cautiously. Although additional nominal associations were identified, none remained significant after correction, suggesting that most allergens are broadly distributed across anatomical locations without strong site-specific patterns. The overall distribution of associations is visualized in Figure 6.

For ease of reference, the principal findings of the study are summarised in Table 6. The table consolidates the key results from the different analyses presented in the Section 3, providing a concise overview of the main observations.

## 4. Discussion

The present study demonstrates that quantitative features derived from the Antera 3D system can be used as inputs for machine-learning models for patch-test assessment. Model performance was satisfactory (AUC = 0.8611) and comparable to previously reported AI-based approaches for patch-test assessment [5,6]. Recent AI-based studies have mainly focused on the analysis of patch-test images using deep-learning models [4,5,6]. In the present study, an alternative methodological approach was adopted by using quantitative measurements derived from Antera 3D imaging as model inputs. The methodology combines quantitative skin features with patient-level validation, temporal Δ-features describing the evolution of skin reactions between 48 and 72 h, SHAP-based model interpretation, and robustness analyses for class-imbalance handling. This approach extends previous image-based studies through the integration of quantitative skin measurements, temporal information, and interpretable machine-learning methods, supporting the use of quantitative imaging data in patch-test assessment. At the same time, comparisons with other AI-based studies in dermatology should be interpreted with caution. Most of these studies have focused on image classification tasks using convolutional neural networks and have been developed using different patient populations, outcome definitions, and validation strategies [6]. In contrast, the present study addressed the evaluation of patch test reactions using quantitative features derived from Antera 3D imaging. Although image-based approaches may identify complex visual patterns, the use of measurable biophysical parameters provides a clearer understanding of how predictions are generated. Therefore, these approaches should be viewed as complementary rather than directly comparable.

A detailed comparison between the present study and recently published AI-based approaches is provided in Supplementary Table S6. Among the recently published AI studies, Vezakis et al. [5] were the first to evaluate deep learning using multimodal Antera 3D images, demonstrating that combining complementary imaging modalities and context-retaining preprocessing improved automated patch-test classification. Ravishankar et al. [19] subsequently demonstrated that a convolutional neural network trained on conventional clinical photographs could accurately discriminate reactive from non-reactive patch-test sites (AUC = 0.940; accuracy = 90.1%). More recently, Kim et al. [20] reported an accuracy of 98.3% using a YOLOv5x object-detection framework trained on a substantially larger dataset of standardized patch-test photographs. Although these studies differ in network architecture and image acquisition protocols, they share a common image-based deep-learning framework that provides limited insight into the quantitative factors contributing to model predictions.

Unlike these image-based approaches, the present study utilized quantitative bioengineering measurements derived from Antera 3D imaging rather than raw images. This enabled direct modelling of clinically relevant parameters describing erythema, haemoglobin distribution and skin morphology, while also allowing incorporation of temporal changes between the 48-h and 72-h patch-test readings. Furthermore, SHAP analysis identified the quantitative variables contributing most strongly to model predictions, providing a level of interpretability that is not inherently available in conventional convolutional neural network models.

The recent review by Burli et al. [21] highlighted the diagnostic challenges of patch testing in patients with skin of colour and emphasised the need for more objective assessment methods. Similarly, the systematic review by Al-tharwane and Al Abadie [22] concluded that although AI-assisted patch testing consistently demonstrates promising diagnostic performance, future research should prioritise external validation, methodological standardisation and explainable AI. The methodology adopted in the present study addresses several of these recommendations by incorporating quantitative imaging biomarkers, patient-level validation, leakage-controlled model development, and explainable machine learning within a clinically interpretable framework.

Based on SHAP analysis, the visual features assessed by the clinician are mapped to measurable parameters, such as CIELAB redness indices (C_a aver*, C_a*) and hemoglobin-related parameters (R_Aver, R_Max, R_Var) (Table 1), which clarifies how the model’s predictions were derived. In particular, in case of a positive result, from the model, it seems to be related to specific changes in the intensity and distribution of erythema. Unlike models based solely on images, which mainly manifest where the model “focuses”, the present approach is more efficient towards a specific diagnostic decision.

One of the key findings of the study is that the progression of reactions over time plays an important role in diagnosis. The model that used changes between the 48 and 72 h intervals (Δ = 72 h − 48 h) performed best (AUC = 0.902), outperforming models based only on measurements from a single time point. This indicates that it is not enough to examine at single temporal measurements, but also it could probably be more effective to examine allergic reactions over time. This finding makes clinically sense: allergic contact dermatitis usually develops gradually, with increasing erythema, edema, and infiltration, while irritant reactions tend to decrease over time. By capturing these changes, the model can better distinguish allergic from non-allergic reactions.

To the best of our knowledge, this is the first study to approach the temporal dynamics of patch tests, which indicates that this information can improve performance as compared to models based on a single time point. However, the temporal difference Δ(72 h − 48 h) should currently be interpreted as a marker of the temporal evolution of the skin response rather than as evidence of an independent construct. Although clinicians performed routine assessments at both 48 and 72 h, the present study was not designed to directly compare temporal delta features with serial clinician evaluations. Therefore, it remains unclear whether these temporal changes provide information beyond that already incorporated into routine clinical assessment or whether they primarily reflect the same temporal patterns recognised by clinicians during evaluation. Future studies directly comparing temporal delta features with serial clinician assessments and biophysical parameters derived from Antera 3D imaging will be necessary to determine their incremental value beyond routine clinical assessment and to clarify their potential role in supporting clinical decision-making.

Without any balancing method, the model manifested a higher AUC and specificity, but sensitivity was low (37.3%), which limits its practical utility. By implementing SMOTE and category weighting, sensitivity improved significantly (67.2%), with a relatively small decrease in specificity. This is clinically relevant, because in this case it is more important to identify true allergic reactions than to simply maximize overall accuracy. Although there are reservations about using synthetic oversampling methods, such as SMOTE, in clinical populations [23,24], our results show that SMOTE can provide tangible benefits when applied in a well-controlled training setting. The decrease in specificity is expected but may be acceptable in settings where avoiding missed positive cases is considered a priority.

A different classification threshold was also tested, in order to better identify positive cases. With this change, the sensitivity increased to 94.9%, while false negatives were reduced from 58 to 9 in the total number of tests. The specificity was reduced, as expected, but this is acceptable in screening scenarios, where it is more important not to miss positive cases. In any case, the model does not replace the healthcare professional, but it can potentially assist in the assessment, especially for the more borderline cases [2] giving a more solid basis for the diagnosis.

The present study also revealed some clinically useful associations between allergens and localization. The most striking finding was the association of 2-hydroxyethylmethacrylate (HEMA) with occupational dermatitis, which remained statistically significant even after correction for multiple comparisons. This is in line with recent data, as exposure to HEMA has increased, mainly due to its use in nail products and dental materials [25,26]. This finding suggests that such approaches can help identify patterns that have practical relevance and may also be useful at the public health level.

At the same time, the value of combining imaging data with machine learning methods to extract useful clinical information is highlighted. In this context, the proposed system can function as a support tool in clinical practice. The data from Antera 3D and the model predictions (AUC = 0.861, 95% CI: 0.830–0.888) can complement the classic ICDRG assessment. By combining measurements, predictions and explanations (as with SHAP), the system can aid in decision-making and reduce cross-examiner differences, especially in more doubtful cases, without replacing the expert’s judgment.

To the best of our knowledge, the present study showed that the use of measurable features combined with interpretable models can help in the evaluation of patch tests. The addition of temporal evolution improved performance, while SHAP analysis provided a better understanding of how predictions are related to clinical features. Overall, the results suggest that a more quantitative approach, which also takes into account time evolution, can be helpful for the specialist, especially in more borderline or doubtful cases.

### 4.1. Perspectives

The present work does not introduce algorithmic novelty; rather, it contributes a clinically grounded, leakage-controlled measurement-and-modelling framework for objective patch-test quantification, together with the first quantitative evidence that reaction kinetics (Δ = 72 h − 48 h) carry an independent predictive signal beyond single-time-point morphology. Specifically, incorporating Δ-features increased the test AUC from 0.861 (95% CI 0.8301–0.8880) to 0.902, with sensitivity reaching 96.8% and specificity of 50.5% at the operating threshold (*t* = 0.22), corresponding to only 3 false negatives among 93 positive samples in the test subset. This represents a clinically meaningful improvement for a screening-oriented task, where missed allergens may have direct consequences for patient management. Furthermore, the proposed framework is, to the best of our knowledge, the first patient-level machine-learning pipeline operating directly on 41 quantitative Antera 3D bioengineering features for ACD patch-test grading, complementing the inherently subjective ICDRG-based assessment—whose inter-observer variability is well documented [2,27] with reproducible biophysical measurements. In addition, temporal Δ-features describing changes between the 48- and 72-h readings were introduced to capture reaction kinetics. By quantifying these temporal changes, the proposed model incorporates information beyond single-time-point measurements, contributing to the improved predictive performance observed in this study.

Through this work, we have introduced a leakage-controlled evaluation protocol uncommon in dermatology ML. Parameters such as GroupKFold and GroupShuffleSplit enforce patient-level partitioning, guarded by a programmatic assertion in code (assert overlap = 0, phase2_modeling.py:149); target-revealing columns are removed at the data-preparation stage; and SMOTE is applied strictly inside the sklearn Pipeline so resampling never touches held-out folds.

Finally, we have introduced a three-way ablation of class-imbalance handling paired with Bonferroni and Benjamini–Hochberg correction for the allergen × location multiplicity, and bootstrap 95% confidence intervals (*n* = 2000) for AUC, sensitivity, and specificity (Section 2.8)—rarely reported jointly in the literature. Along with that, SHAP-based clinical interpretability identifying C_a* aver, R_Var, C_a*, R_Aver, and R_Max were identified as dominant predictors, with each linked back to erythema and micro-relief physiology. Thus, the novelty of this study is therefore translational and methodological rather than algorithmic.

### 4.2. Methodological Considerations

To evaluate the impact of synthetic oversampling on model performance, SMOTE was assessed through a predefined robustness analysis rather than adopted as a default preprocessing strategy. To minimize the risk of optimistic performance estimates commonly associated with synthetic oversampling in medical machine learning [28], SMOTE was applied exclusively within the training folds by embedding the resampling procedure in an imbalanced-learn pipeline, ensuring that synthetic observations were never introduced into the validation folds or the independent test set. Furthermore, all data partitioning and model evaluation were performed at the patient level using GroupKFold and GroupShuffleSplit (Section 2.3), ensuring that observations from the same patient were never included in both training and evaluation datasets. Three balancing strategies were evaluated under identical patient-level cross-validation and bootstrap confidence intervals (*n* = 2000): (i) SMOTE combined with balanced class weights, (ii) balanced class weights alone, and (iii) no balancing (Figure 2). Model generalization was subsequently assessed using both five-fold patient-level cross-validation and an independent held-out test set.

The comparison of the three balancing strategies (Figure 2) demonstrated a clinically relevant trade-off between discrimination and minority-class detection. The no-balancing configuration achieved the highest discrimination (Test AUC = 0.889; CV AUC = 0.880 ± 0.016), but at the expense of markedly reduced sensitivity (37.3%), indicating that nearly two-thirds of true positive patch-test reactions would have been missed. Applying balanced class weights alone improved sensitivity to 61.6% while maintaining good discrimination (Test AUC = 0.878; CV AUC = 0.873 ± 0.017). The combination of SMOTE and balanced class weights further increased sensitivity to 67.2%, with Test AUC = 0.861, specificity = 86.1%, and CV AUC = 0.869 ± 0.017. Although this configuration produced a modest reduction in AUC, it provided the most appropriate balance between sensitivity and specificity for a screening-oriented clinical decision-support tool, where failure to detect a true allergic reaction may result in continued exposure to an unidentified allergen, whereas false-positive results can subsequently be clarified through routine confirmatory clinical assessment. 

In addition to sensitivity and specificity, the positive predictive value (precision) of the selected configuration was moderate (0.515 on the independent test set and 0.511 under five-fold cross-validation), indicating that approximately half of the sites flagged as positive corresponded to confirmed reactions. This finding is expected in the present setting for two reasons. First, positive reactions were relatively infrequent (approximately 17% of observations); under such class imbalance, even a well-discriminating model (AUC = 0.861) may achieve only moderate precision because of the well-known base-rate effect, while still maintaining a high specificity (0.861). Second, the operating point was deliberately selected to favor sensitivity, since in a screening context missing a true reaction (false negative) is clinically more consequential than generating a false positive, which can subsequently be resolved through routine confirmatory assessment. Accordingly, the model is intended as a screening aid that complements, rather than replaces, expert ICDRG reading, prioritizing the detection of genuine reactions over maximizing precision. The robustness of the selected approach was further supported by the close agreement between cross-validation performance (AUC = 0.869 ± 0.017) and the independent test-set performance (AUC = 0.861; 95% CI 0.830–0.888, bootstrap *n* = 2000), suggesting that synthetic oversampling did not result in meaningful overfitting or inflated performance estimates.

Random Forest was selected as the final classifier following an empirical comparison with Logistic Regression and a multilayer perceptron (MLP). These algorithms were chosen to represent three complementary modelling paradigms, namely tree-based ensemble learning, linear classification, and neural-network approaches. Random Forest achieved the best overall balance between discrimination, sensitivity, and specificity (Table 3). The MLP was included as a benchmark neural-network model to evaluate whether a deep-learning architecture would provide an advantage on this medium-sized tabular dataset; however, its performance was inferior to that of Random Forest (Test AUC: Random Forest = 0.861, Logistic Regression = 0.844, and MLP = 0.836). Accordingly, Random Forest was selected as the final model on the basis of its empirical performance and clinical utility rather than a priori methodological preference. This pattern is consistent with Grinsztajn et al. (2022), who showed that tree-based models outperform deep-learning methods on medium-sized tabular datasets [29].

Regarding other machine-learning algorithms, the comparison presented in Table 3 spans three major modelling paradigms. In addition, KNN was evaluated using GridSearchCV, while XGBoost was investigated for the severity regression task.

### 4.3. Limitations

This study has some limitations that should be considered. First, although the design was prospective and validated at the patient level, the study was conducted at a single center, which may affect the generalizability of the results. Future validation using independent cohorts from other institutions will be important to confirm the robustness and generalisability of the proposed approach. In addition, although predefined eligibility criteria were applied before data analysis, no formal comparison was performed between included and excluded individuals; therefore, some degree of selection bias cannot be entirely excluded. Secondly, although the dataset included 4477 observations, these were derived from 194 patients, which may limit the variety of clinical presentations [30]. This may also have influenced the allergen–location analyses, where only one association remained significant after correction for multiple testing. It is possible that the limited number of patients reduced the ability to detect additional associations. Therefore, larger studies are needed to further explore potential relationships between allergen sensitisation and anatomical location.

Another limitation is that the model was trained using ICDRG scores, which are inherently subject to some degree of observer variability. The aim of the proposed approach is therefore not to replace expert assessment but to support it in a more standardised manner. As ICDRG assessments performed by experienced dermatologists served as the reference standard, the present study does not allow a direct comparison between model performance and dermatologist performance. Future studies incorporating independent reference standards, such as histopathology or the Repeated Open Application Test (ROAT), as well as blinded assessments by multiple clinicians, may further strengthen label validation and help determine how such tools can complement routine clinical assessment and improve inter-observer consistency. In addition, evaluating model performance independently of the initial labels may help clarify its potential for more objective classification. Finally, our approach was based on the Antera 3D imaging system and it is not yet clear whether the results can be applied to other devices. Although the use of temporal evolution improved performance, the analysis was limited to two patch test reading time points (48 and 72 h) and relied on Δ-feature representations rather than dedicated longitudinal models. Larger cohorts with denser temporal sampling could enable the evaluation of more sophisticated time-series approaches and provide a more complete picture of the evolution of patch test reactions. It also remains uncertain whether the temporal information captured by the Δ-features adds information beyond that already considered by clinicians during routine patch test readings, or simply reflects the same changes in skin responses. Studies combining serial clinical assessments with objective Antera 3D measurements may help address this question.

Finally, SMOTE interpolates within the observed minority distribution and cannot create genuinely novel phenotypes, so external validation on an independent cohort remains necessary.

## 5. Conclusions

The present study investigated a different approach for the evaluation of patch tests, based on measurements from the Antera 3D system combined with machine learning methodologies. The proposed model showed satisfactory performance (AUC = 0.861) and, which with threshold adjustment, achieved high sensitivity (94.9%). One of the key points, of our study, was the discovery that when temporal progression was considered the predictive potential of our model improved greatly. This shows that a single measurement is probably not adequate, as well as temporal progression plays a role. In other words, the course of the reaction over time seems to have substantial diagnostic value in allergic contact sensitization. SHAP analysis showed that the model relies mainly on features that have direct clinical relevance, such as the intensity and distribution of erythema. This helps to better understand how the predictions are derived. At the same time, a relationship was observed between HEMA and occupational dermatitis, which is consistent with exposure to specific allergens. Overall, the results indicate that a more objective approach can help in the evaluation of patch tests and reduce inter-examiner differences, supporting clinical practice.

## Figures and Tables

**Table 1:** Selected biomedical features for color analysis (CIELAB: L*, a*, b*) and for the assessment of skin redness (erythema) based on hemoglobin-related indices.

	Variable	Description
CIELAB	C_a* aver	Mean value of the a* component (red–green axis) within the selected region.
	C_a*	Red–green color component of the CIELAB color space.
	C_b*	Yellow–blue color component of the CIELAB color space.
	C_ITA	Individual Typology Angle (ITA°), used for skin tone classification.
	C_b*aver	Average value of the CIELAB b* coordinate, representing the yellow–blue color component.
	C_RHVA (%)	Relative High Variation Area, defined as the percentage of the selected region exhibiting high color variation.
	C_HVA (mm^2^)	High Variation Area, defined as the surface area within the selected region exhibiting high color variation.
	C_Variation	Color variation within the selected region, reflecting the degree of skin color heterogeneity.
	C_L*	Lightness component of the CIELAB color space, representing skin brightness.
Redness (or erythema)	R_Score	Hemoglobin-related skin severity (mild to very severe).
	R_Var	Spatial variation of hemoglobin concentration.
	R_Aver	Average concentration of hemoglobin.
	R_Max	Maximum concentration of hemoglobin.

**Table 2:** Demographic and clinical characteristics (*n* = 194). Continuous variables: mean ± SD. Categorical: number (%).

Characteristic	Value (*n* (%))
Female	155 (79.9%)
Male	39 (20.1%)
Age (mean ± SD)	41.9 ± 13.7
Non-smokers	113 (56.5%)
Smokers	68 (34.0%)
Former smokers	13 (6.5%)
Atopic dermatitis (AD)	80 (41.2%)

**Table 3:** Model Comparison: Random Forest vs. Logistic Regression vs. MLP (* Asterisk depicts a statistical significance of *p* < 0.05).

Model	Test A.	Sensitivity	Specificity	CV AUC
Random Forest	0.8611 *	0.6723 *	0.8610	0.8691 ± 0.017
Logistic Regression	0.8437	0.6158	0.9069 *	0.8729 ± 0.017 *
MLP (Deep Learning)	0.8365	0.6560	0.8620	0.8258 ± 0.020

**Table 4:** Aggregated confusion matrix from the patient-level five-fold cross-validation on the training set (3494 observations), pooled over the five held-out validation folds.

	Predicted Negative	Predicted Positive
Actual Negative	2.482 (TN)	412 (FP)
Actual Positive	170 (FN)	430 (TP)

**Table 5:** Comparison of different time combinations for predicting allergic reactions with Random Forest, with basic sample sizes and performance indicators (Numbers in bold indicate optimal parameters combination).

Dataset	*N*	Positive (%)	CV AUC (mean)	CV AUC (std)	Test AUC	Sens. (*t* = 0. 50)	Spec. (*t* = 0. 50)	Sens. (*t* = 0. 22)	Spec. (*t* = 0.22)
Combined (all)	4477	17.4%	0.869	0.017	0.861	67.2%	86.1%	91.5%	57.8%
48 h Only	1413	20.7%	0.818	0.034	0.835	65.6%	86.2%	90.6%	57.9%
72 h Only	1530	31.7%	0.889	0.017	0.838	63.8%	88.6%	85.7%	60.7%
48 h + 72 h (no baseline)	2943	26.4%	0.841	0.026	0.829	65.6%	81.2%	93.5%	47.7%
**72 h + Temporal Deltas**	**1411**	**32.0%**	**0.883**	**0.032**	**0.902**	**71.0%**	**86.2%**	**96.8%**	**50.5%**

**Table 6:** Summary of key results from the main analyses.

Category	Key Finding
Cohort characteristics	194 patients and 4477 observations were included in the final analysis.
Model comparison	Random Forest showed the highest test discrimination (AUC = 0.861) among the evaluated models.
Class imbalance handling	The Random Forest model combining SMOTE and class weighting was selected as the final model.
Threshold optimisation	Lowering the decision threshold increased sensitivity from 67.2% to 94.9%, reducing false negatives.
Model interpretability	SHAP identified erythema-related features (R_Aver, R_Max, R_Var) and a*-related colour descriptors (C_a*, C_a* aver) as the most influential predictors.
Temporal dynamics	Incorporation of temporal features improved diagnostic performance (AUC = 0.902).
Allergen–location associations	Only the association between HEMA and occupational dermatitis remained significant after Bonferroni correction.

## Data Availability

Data are available upon reasonable request.

## Supplementary Materials

The following supporting information can be downloaded at: https://www.mdpi.com/article/10.3390/bioengineering13070818/s1, Figure S1a: Decision Curve Analysis; Figure S1b: Decision Curve Analysis with Confidence Intervals; Figure S2: SHAP Comparison Importance; Figure S3: SHAP Baseline Summary; Figure S4: SHAP Temporal Summary; Table S1: RandomSeed Sensitivity; Table S2: SOTA Comparison; Table S3: Feature Selection Ablation; Table S4: SHAP PerFold Ranking; Table S5: SHAP Ranking Comparison; Table S6: Comparison of the present study with recent AI-based approaches for patch-test assessment.
