# Supplementary material for: Quantitative Evaluation of Patch Test Reactions Using a 3D Camera-Derived Features and Machine Learning: The Role of Temporal Dynamics
Source: Bioengineering (Basel). 2026 Jul 16;13(7):818. doi: 10.3390/bioengineering13070818 (PMC13404982; doi:10.3390/bioengineering13070818)
Supplement: Supplementary file 1 [file bioengineering-13-00818-s001.zip › Table_S6_.pdf]

**Table S6. Comparison of the present study with recent AI-based approaches for patch-test assessment.**

| Study | Modality / data | AI / method | Task | Validation | Reported performance |
|---|---|---|---|---|---|
| Present study | Quantitative Antera 3D bioengineering features (CIELAB colour, haemoglobin, volumetric and morphological parameters) with temporal Δ48–72 h features; tabular dataset (194 patients, 4,477 observations) | Random Forest + SMOTE (training folds only) + SHAP explainability | Binary classification of positive patch-test reactions (ICDRG >0) | Patient-level GroupKFold (k = 5) and independent held-out test set (GroupShuffleSplit 80/20) | Test AUC 0.861 (sensitivity 0.672, specificity 0.861, precision 0.515); best temporal configuration AUC 0.902 |
| Ravishankar et al. 2024 [19] | Clinical photographs (raw RGB images); 13,622 images from 125 patients | Convolutional Neural Network (CNN) | Binary classification of reaction vs non-reaction | Validation strategy not explicitly reported | AUC 0.940; accuracy 90.1%; sensitivity 86.0%; specificity 90.2% |
| Kim et al. 2025 [20] | Standardised clinical photographs; 83,629 training images, 1,312 evaluation and 1,536 validation images | YOLOv5x object detection | Ordinal erythema grading (scores 0–4) | Independent evaluation and validation datasets | Accuracy 0.983; F1-score 0.982; AUCs of 0.914 (grade 0), 0.838 (grade 1) and 0.865 (grade 2). |
| Vezakis et al. 2023 [5] | Multimodal Antera 3D skin images (colour, haemoglobin/redness, texture, fine lines, folds and volumes); 1,579 images from 200 patients | EfficientNet-B0 CNN with context-retaining image pre-processing | Binary classification of positive patch-test reactions | Five-fold cross-validation | Best-performing modality achieved >86% recall and 94% specificity; accuracy ≈0.91 and F1 ≈0.87 |
| Burli et al. 2023 [21] | Literature review of patch testing in patients with skin of colour | Narrative review | Clinical interpretation of patch tests in skin of colour | n/a | No AI model or original performance metrics |

| Study | Modality / data | AI / method | Task | Validation | Reported performance |
|---|---|---|---|---|---|
| Al-tharwane & Al Abadie 2026 [22] | Systematic review of AI applications in patch testing | Systematic review (PRISMA) | Critical appraisal of AI methods in patch testing | n/a | Reported that published CNN studies achieved accuracies ranging from 90.1% to 99.5%, while emphasising limited external validation and dataset heterogeneity |
