# Supplementary figures and images for: Quantitative Evaluation of Patch Test Reactions Using a 3D Camera-Derived Features and Machine Learning: The Role of Temporal Dynamics
Source: Bioengineering (Basel). 2026 Jul 16;13(7):818. doi: 10.3390/bioengineering13070818 (PMC13404982; doi:10.3390/bioengineering13070818)

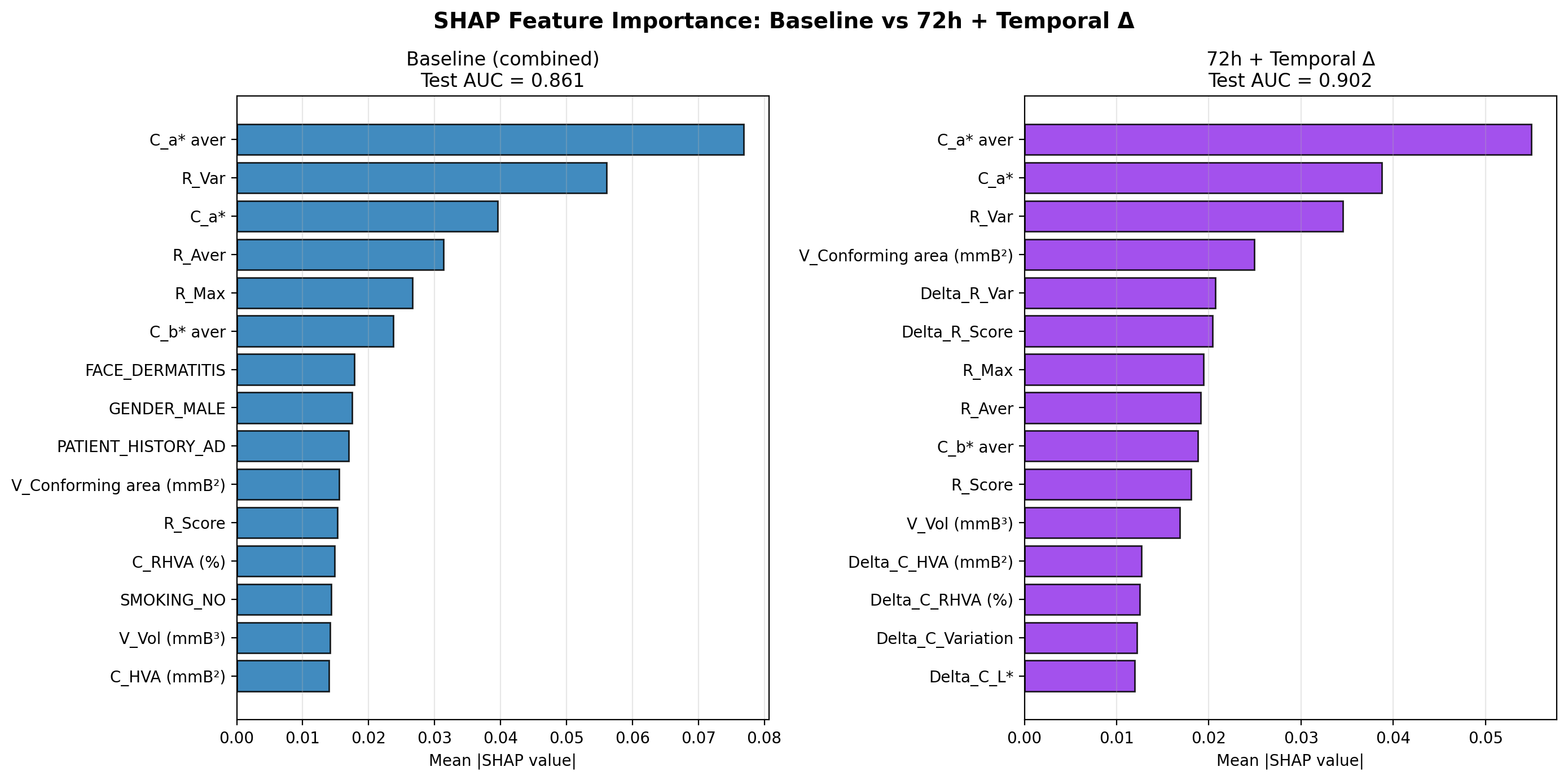

Supplement: Supplementary file 1 [file bioengineering-13-00818-s001.zip › Fig. S2-SHAP Comparison Importance.png]

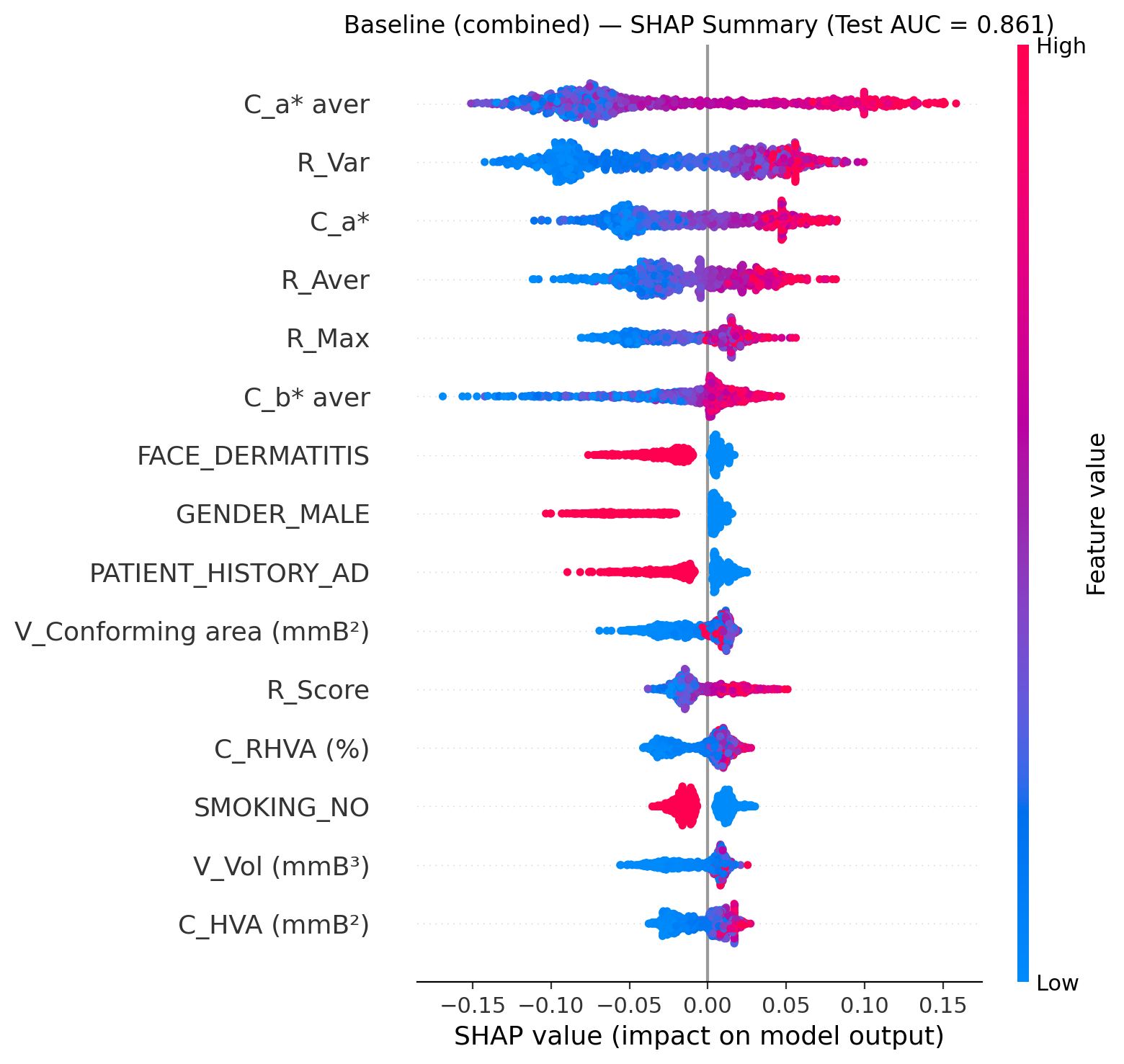

Supplement: Supplementary file 1 [file bioengineering-13-00818-s001.zip › Fig. S3-SHAP Baseline Summary.png]

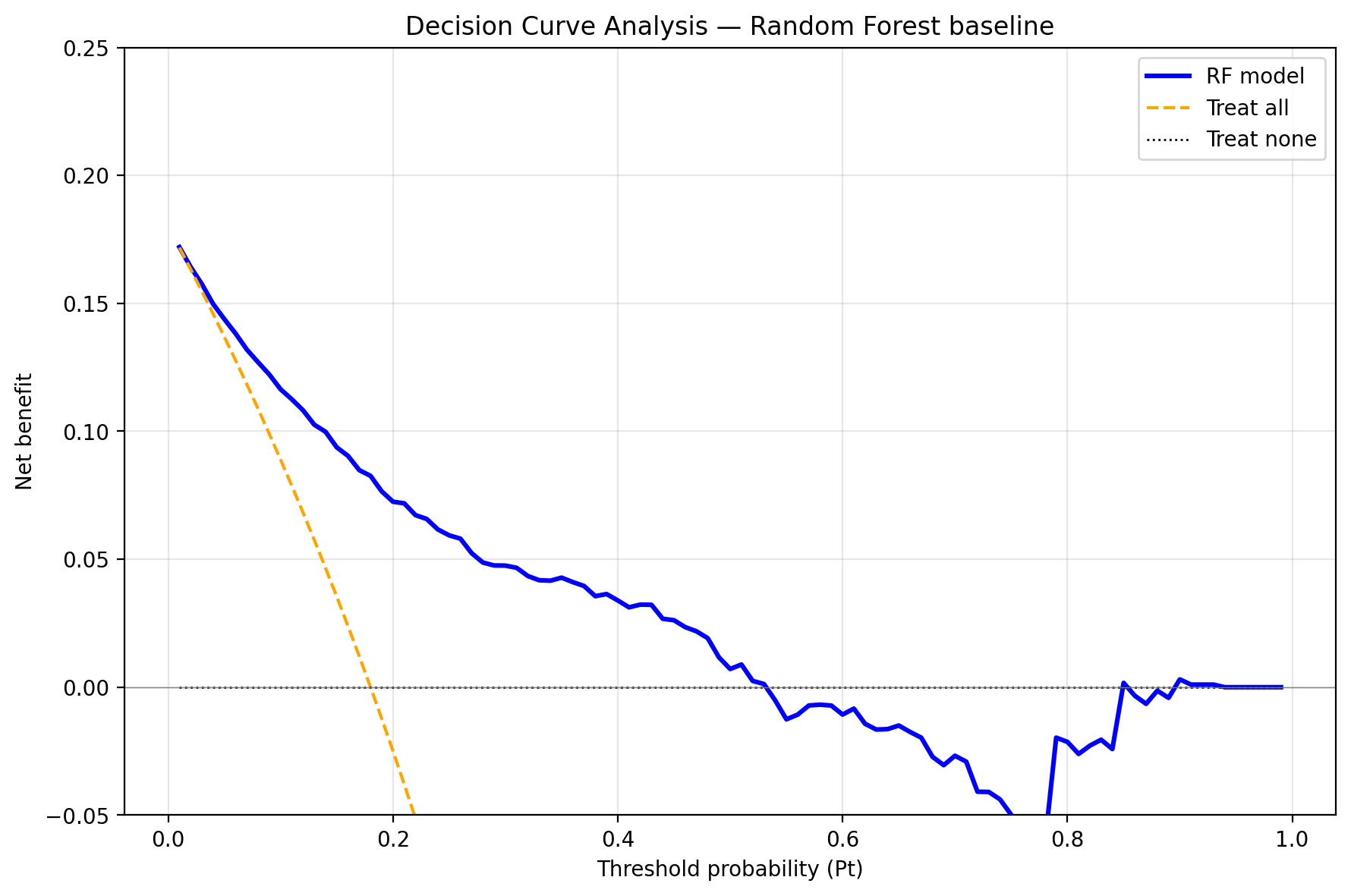

Supplement: Supplementary file 1 [file bioengineering-13-00818-s001.zip › Fig_S1a_DecisionCurveAnalysis.png]

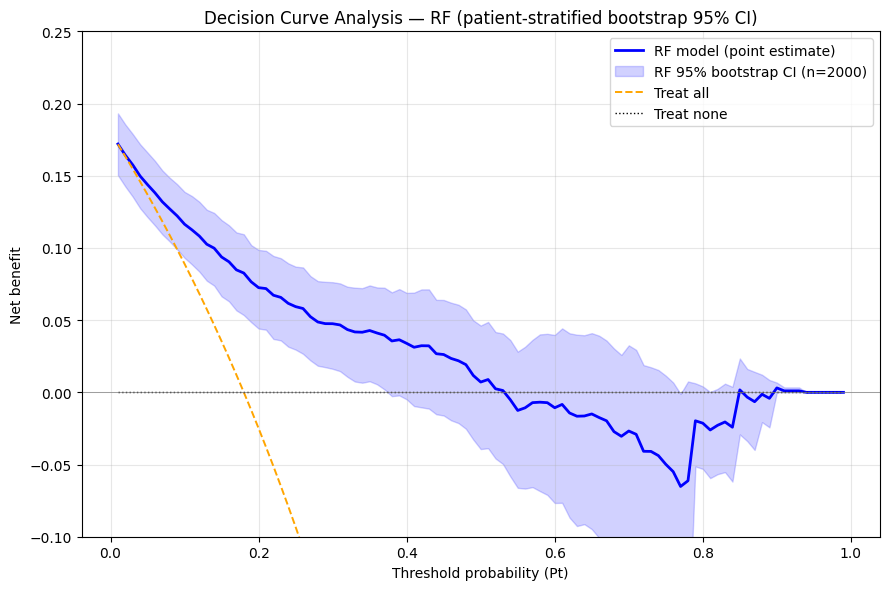

Supplement: Supplementary file 1 [file bioengineering-13-00818-s001.zip › Fig_S1b_DecisionCurveAnalysis_with_CI.png]

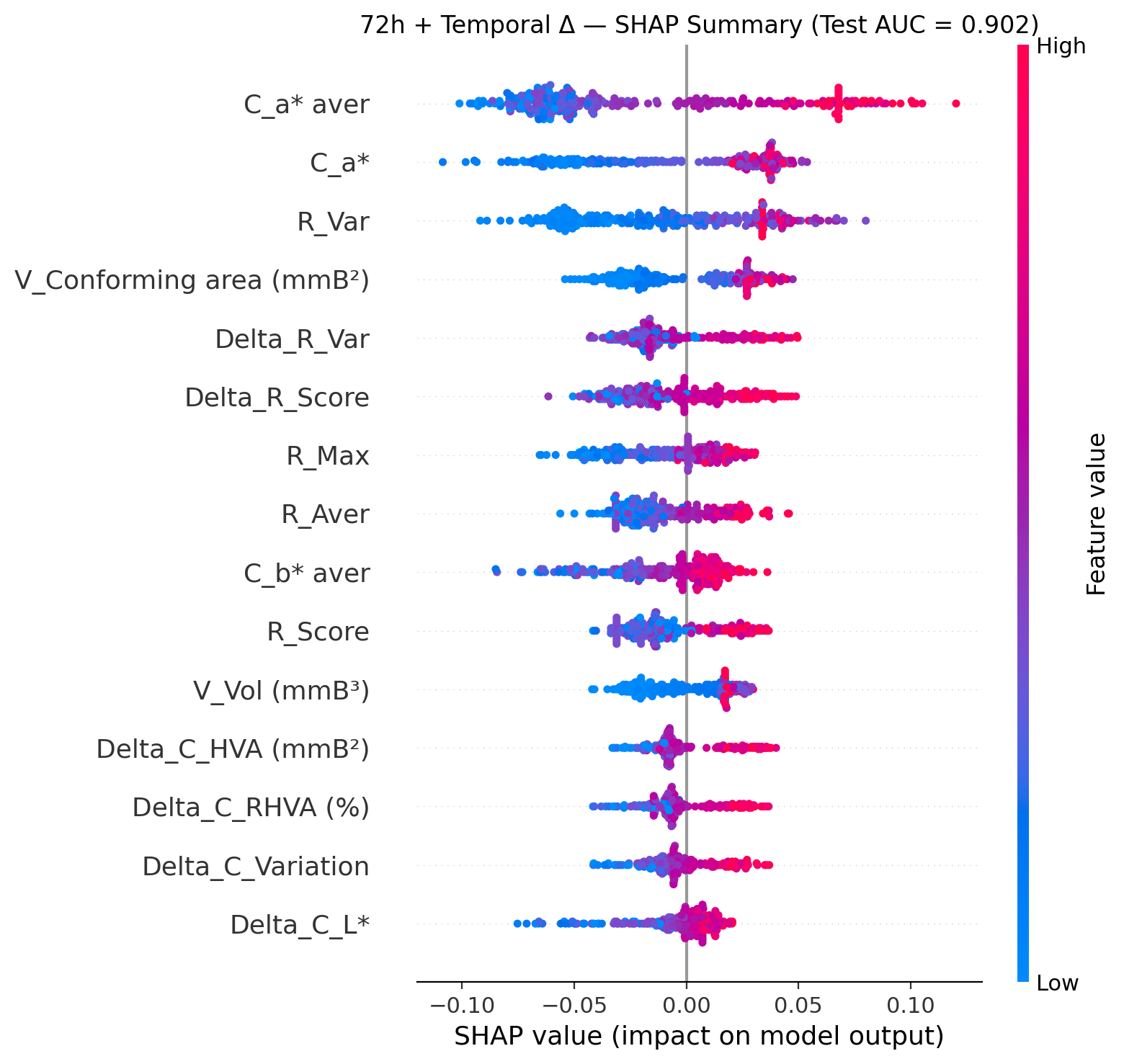

Supplement: Supplementary file 1 [file bioengineering-13-00818-s001.zip › Fig_S4_SHAP_Temporal_Summary.png]
